# Every scientist is a memory researcher: Suggestions for making research more memorable

**DOI:** 10.12688/f1000research.6053.1

**Published:** 2015-01-22

**Authors:** Christopher R. Madan

**Affiliations:** 1 Department of Psychology, University of Alberta, Edmonton, Alberta T6G 2E9, Canada; 2 Department of Psychology, Boston College, Chestnut Hill, MA 02467, USA

**Keywords:** scientific writing, memory, citation frequency, scholarly communication, publishing

## Abstract

Independent of the actual results, some scientific articles are more memorable than others. As anyone who has written an article collaboratively knows, there are numerous ways a manuscript can be written to convey the same general ideas. To aid with this, many scientific writing books and editorials provide advice, often anecdotal, on how to make articles more memorable. Here I ground these suggestions with empirical support from memory research. Specifically, I suggest that researchers consider how to emphasize their work’s novelty, strive to describe their work using concrete, easy-to-understand terms, and use caution when attempting to evoke an emotional response in the reader. I also discuss considerations in title selections and conference presentations.

## Introduction

Every scientist wants their paper to be read by their peers, their theories and results to be remembered, and their work to be cited in subsequent papers. Many factors influence the likelihood that a paper will be read and later cited by a reader, such as the relevance of the topic to the reader’s own interests, the journal that the work was published in, the quality of the experimental design (e.g., sample size, statistical rigor), and the work’s exposure in the media and social media (i.e., altmetrics) ( Bartneck & Hu, 2009; Callaham *et al.*, 2002; Eyre-Walker & Stoletzki, 2013; Fraley & Vazire, 2014; Leimu & Koricheva, 2005; Piwowar, 2013). (While the impact factor of the journal an article is published in influences the likelihood of the work being read by other researchers, the citation frequency of the work itself is definitively a more relevant measure of an article’s impact, e.g., Brembs *et al.*, 2013; Editorial, 2003; also see “Measuring the Impact of Scientific Articles” by Peterson). In addition to these factors that are more generally acknowledged as influencing citation frequency, other factors also correlate with citation frequency, such as gender, number of co-authors (particularly those from other institutions), and citation networks (i.e., research groups that often cite each other) ( Borsuk *et al.*, 2009; Campbell *et al.*, 2013; Maliniak *et al.*, 2013). In addition to these factors, other factors can further influence citation frequency, such as *memorability*. Though memorability may co-vary with some of the other factors mentioned, specific strategies, grounded in the human memory literature can be used to make a research paper more memorable.

A paper’s memorability is more difficult to quantify than many other factors; however, it could be viewed as even more important since the researcher may have more control over it. A researcher should not change their research topic to a ‘hotter’ topic simply for the sake of garnering more citations, nor should they seek out co-authors from other institutions solely in the hopes of garnering more citations. However, there is often more than one way to write a paper on a given research study, depending on how the topic is related to the relevant background literatures. It is also possible to make a paper more readable by using more direct language when describing the experimental design and results (see Dunleavy, 2003; Pinker, 2014; and Kail, 2014, for detailed discussions of scientific writing). However, the focus of the current paper is on grounding anecdotal scientific writing advice in the human memory literature, providing empirical support for making a paper more memorable. (I should state that my papers likely have not capitalized on these memory principles as well as they should have—connecting scientific writing to memory findings has been a learning experience for me as well). Many of these scientific writing suggestions and related memory findings are listed in Table 1.

**Table 1.** Summary of scientific writing advice and related memory findings.

Scientific Writing Advice	Related Memory Finding
General Scientific Writing	
First-mover advantage	primacy, novelty, distinctiveness
Multidisciplinary	novelty, distinctiveness
Clear and concise language	concreteness
Engaging prose	emotional arousal
Strong endings	peak-ends rule, end-anchor
Title Selection	
Concise	word length
Informative	concreteness
Question	elaborative processing
Catchy/attention commanding	emotional arousal
Not amusing	emotional arousal
Pleasantness	emotional arousal
Specific to Poster and Oral Presentations	
Layout, colors, readability	concreteness
Big picture ideas	elaborative processing
Figures	picture superiority, bizarre imagery, distinctiveness

## Be novel

One of the most robust and well-known findings in the memory literature is the serial position curve ( Ebbinghaus, 1885; Hasher, 1973; Murdock, 1962). Briefly, if a list of items is presented to participants and they are subsequently asked to recall all of the items from the list that they can, the participants are more likely to recall the first and last items in the list, relative to the intermediate items. These two effects are respectively referred to as the primacy and recency effects. With respect to citation frequency, Newman (2009) has reported a “first-mover advantage”, suggesting that a researcher publishing a modest paper on next year’s hot topic will accrue more citations than if they had instead published an outstanding paper on the current year’s topic. This difference in citation frequency could be attributed to a primacy effect, where other researchers are more likely to remember the first paper on a topic because it was the *first* and is thus more easily sampled from memory when thinking of seminal studies of a topic. Furthermore, the first paper published on a topic is more *novel* than its contemporaries. A large body of memory research supports this notion of memory enhancement for novel information ( Gabrieli *et al.*, 1997; Wittman *et al.*, 2007).

Another way of viewing effects of novelty on memorability is that novel information is more *distinctive*. While other studies may demonstrate modest advances in our understanding of a topic, novel studies stand out and take the field forward more significantly. The finding that distinctive information is remembered better is known as the isolation effect or the von Restorff effect ( von Restorff, 1933). In an experimental setting, the von Restorff effect is often studied by presenting participants with a list of words sequentially, with one word being more distinct in a perceptual (e.g., font color, font size) or conceptual (e.g., emotional, semantic category) dimension. In terms of scientific writing, a researcher likely reads many papers over the course of several months, and one that is distinctive and stands out from the rest is more likely to be subsequently remembered and later cited when the researcher is writing their own manuscript.

While memory findings can help explain why novel research is likely to be remembered and cited, making a study appear novel is not as straightforward. Some researchers are quite adept at discovering new advances in their fields, such as by integrating ideas across disciplines, and the resulting products are often novel. Indeed, analyses of high-impact papers have found that multidisciplinary teams are more likely to produce novel and innovative advances ( Uzzi *et al.*, 2013). For some guidance on coming up with novel research ideas, see Yewdell (2008).

## Be interesting

Another common suggestion for scientific writing is to make the paper ‘interesting’ (e.g., Bartunek *et al.*, 2006; Davis, 1971; Gray & Wegner, 2013; Sand-Jensen, 2007). Gray & Wegner (2013) suggest six guidelines for making one’s research interesting. While summarizing their eloquently described guidelines does them a disservice, briefly, they make three suggestions related to choosing the research question and three related to the how the researcher goes about answering the question. They suggest that a researcher choose a research question that (1) focuses on the phenomena of interest first, rather than extant studies and theories; (2) is surprising, study phenomena that are counter-intuitive (“If results were exactly as predicted, would they be interesting”?); and (3) would interest a layperson and not just researchers (but note: “Conversely, countering laypeople’s intuitions may yield fewer immediate citations, especially if the research does not easily fit into established scientific paradigms”). On the other side, they suggest that the researcher addresses the research question using an experiment that (4) would be engaging to the participant; and (5) lends itself to simple statistics (“If a more complicated analysis is needed, think about redesigning the study; 4-way interactions can be explained, but would anyone care enough to listen?”). Finally, Gray and Wegner suggest that (6) the paper be written in a way that emphases it’s importance and the universal truths that it is built upon are clear and evident to a layperson, not just researchers. (Also see Bartunek *et al.*, 2006, for an empirical analysis that converged on similar reasons of why an article was found to be interesting by readers).

Gray and Wegner’s guidelines can map on to several memory-relevant mechanisms. The idea of surprise corresponds well with the suggestion of making the work appear novel and distinctive from prior published studies. They also suggest that writing be generally more understandable and engaging. One implication of this suggestion is to describe the motivation and interpretation of the study using more *concrete* and imageable terms, rather than abstract concepts. In this regard, memory studies have definitive evidence that concrete words are remembered better than abstract words ( Paivio, 1971; Paivio, 1986). Evidence also shows that imageability enhances association learning ( Madan *et al.*, 2010), which may make it easier to remember the links between different ideas within the paper later on. Providing illustrations of concepts (e.g., hypothesized results; theoretical models) may also help in this regard.

The notion that writing compelling and engaging prose would increase memorability could also be thought of as adding an *emotional arousal* component. Emotional content itself is often better remembered ( Kensinger & Corkin, 2003; Talmi & Moscovitch, 2004). However, in contrast to imageability, which enhances both item- and association-memory, the effects of emotional arousal on memory appear to be more of a double-edged sword. Specifically, while emotional items (e.g., words, images) tend to be better remembered than neutral items, this often is at the cost of memory for the context that the emotional items are learned in. If the context is intrinsic to the emotional item (e.g., font color), memory for the associated information may be enhanced, however, when the context is more separable, such as two unrelated words, memory for the association is impaired ( Kensinger, 2009; Madan *et al.*, 2012; Mather & Sutherland, 2011). With respect to scientific writing, if an amusing anecdote is used to help motivate the research question, the anecdote—as an emotional item—may be remembered well, but if too unrelated to the actual topic of the paper, the reader may later have trouble recalling where they read the anecdote.

Additionally, Gray and Wegner rightly comment that “People have a powerful memory for endings ( Kahneman *et al.*, 1993), and so you want the reader to remember your paper with a tinge of giddiness and awe”. Indeed, Kahneman *et al.* (1993) found that in patients undergoing a colonoscopy, post-operative pain judgments were correlated with the peak intensity of pain (as judged in real time) and with the pain intensity at the end, but not with the duration of the procedure. Delayed judgments relating to positive experiences, such as vacations, are similarly influenced by peak and end intensities ( Mitchell *et al.*, 1997). This phenomenon, known as the *peak-ends rule*, demonstrates that people’s memories of an experience are biased, with the ending being particularly important.

## Comments on selecting a title

When evaluating an article’s relevance, the reader likely first starts with the title. Studies of citation frequencies have yielded mixed results, with some finding higher citation rates associated with longer titles ( Habibzadeh & Yadollahie, 2010; Jacques & Sebire, 2010), others suggesting the use of shorter titles ( Paiva *et al.*, 2012; Subotic & Mukherjee, 2014), and others yet observed no relationship between title length and citation rates ( Jamali & Nikzad, 2011). Memory research suggests that longer words are harder to remember ( Baddeley *et al.*, 1975), but the title of a research paper is more complex—the string of words that comprise a title have meaning. Along these lines, papers with titles that described the study’s results had higher citation rates ( Jamali & Nikzad, 2011; Paiva *et al.*, 2012). As examples of this, “Articles with short titles describing the results are cited more often” ( Paiva *et al.*, 2012) and “Emotional arousal does not enhance association-memory” ( Madan *et al.*, 2012). However, there is evidence that questions directly describing the research question may have higher citation rates ( Jamali & Nikzad, 2011), e.g., “Is the enhancement of memory due to reward driven by value or salience”? ( Madan & Spetch, 2012). Question-based titles are more likely to engage the reader to think about the research question for themselves, relative to titles that more generally describe the research topic or state the main result. This is particularly important since people remember more about information they think about elaboratively, as found in the *levels-of-processing* framework ( Craik & Lockhart, 1972).

Focusing more on the content, many suggest that a title be ‘catchy’ ( Atkin, 2002; DeBakey, 1977; Schultz, 2009). While this is a good suggestion at face value, one must subsequently consider—what makes a title catchy? Atkin (2002) focused on phrases that suggested innovation, specifically “paradigm shift” and “pushing the envelope”. While these phrases may be catchy, the also should not be over used. Schultz (2009, p. 21) describes the purpose of the title as: “The title is your first opportunity to attract an audience to your paper. A well-worded and catchy title can lure reluctant readers to take a closer look at your paper”. More completely, Schultz suggests that titles should be informative, accurate, clear, concise, and attention commanding (see Schultz, 2009, for further details).

Regardless of one’s opinion of catchy titles, it is important to consider if the potential title is informative ( Comroe, 1966; DeBakey, 1977; Dunleavy, 2003; Eva, 2013; Hartley, 2005; Kazdin, 2013; Mermin, 2003; Paiva *et al.*, 2012; Schultz, 2009; also see “Why do academics and PhDers carefully choose useless titles for articles and chapters?” by Dunleavy). While informative titles tend to be longer, they also are more likely to attract readers who are interested in the topic. As an example, Hartley provides a catchy title that was assigned to an article of his (“More sex please, we’re psychologists”) along with his original title (“Were there any sex differences? Missing data in psychology journals”). Clearly, these titles are not equally informative of the topic of the article, providing an example of when aiming for a catchy title can go too far. If a title is catchy but not informative, it is unlikely to attract readers who are indeed interested in the topic ( DeBakey, 1977). Ideally, catchy titles can still be informative. For instance, “Short and amusing: The relationship between title characteristics, downloads, and citations in psychology articles”. ( Subotic & Mukherjee, 2014) and “Building a memory palace in minutes: Equivalent memory performance using virtual versus conventional environments with the Method of Loci” ( Legge *et al.*, 2012). In many fields, the use of colons in titles has been found to be fairly common, with papers not using colons being cited less often; however, this effect varied greatly between disciplines and was even reversed in some ( Buter & van Raan, 2011).

Several other title properties have also been considered. For instance, a title’s perceived amusement has been found to either have no influence on citation rates ( Subotic & Mukherjee, 2014) or possibly a negative effect ( Sagi & Yechiam, 2008). Pleasantness is positively correlated with citation frequency ( Sagi & Yechiam, 2008), though it is not clear if emotion-memory effects mediate this relationship. Whissell *et al.* (2013) measured trends in titles over the last fifty years and observed shifts towards using more concrete and emotional words in more recent years.

## Presentation-specific advice

While the notions of communicating research such that it is novel and interesting are important regardless of whether a project is written as a manuscript or presented at a conference, some advice is more specific to presenting research. Written firmly tongue-in-cheek, ( Wolcott, 1997a; Wolcott, 1997b) provides many great suggestions for poster and oral presentations. (The original versions of these newsletter articles have been archived online: poster [1997a], oral [1997b]). Briefly, in an aptly titled article, “Mortal sins in poster presentations or, How to give the poster no one remembers”, Wolcott (1997a) suggests poster presenters consider how to logically layout and color the poster, and select an informative title. Most importantly, Wolcott (1997a) provides advice on formatting text to enhance readability, e.g., size, spacing, concise phrasing, organization, and color scheme. See Block (1996) for more poster-related advice. For selecting colors that are distinguishable by color-blind readers, see Wong (2011).

When discussing oral presentations, Wolcott (1997b) gives some similar advice, such as that the content on the slides be easy to read, not overwhelming, and focus on the bigger picture ideas rather than staying too close to the results. Additionally, presenters should make sure to talk to their audience (as opposed to talking at the projector screen), practice the presentation with a friendly audience, be aware of the contents of upcoming slides, and speak sufficiently loudly. See Harolds (2012) and Schultz (2009, especially p. 284) for additional advice for oral presentations.

As evident by Wolcott’s suggestions, presenting one’s research involves additional facets that are present in manuscripts. For instance, text in manuscripts must formatted following a journal’s guidelines, preventing font selection, color, and spacing from being potential issues. Furthermore, manuscripts must follow standard organizational structures (e.g., Introduction, Methods, Results, Discussion). In contrast, both poster and oral presentations involve these additional decisions, which may result in possible missteps. With respect to making your presentation memorable, if the audience is unable to follow along with the presentation, e.g., the presenter is not sufficiently audible or if the slides/poster content distracts rather than serves as a visual aid, then the presentation will be less memorable. Furthermore, other factors that may simply not have crossed the mind of the presenter may also have a significant effect on in-person presentations, but be irrelevant to manuscripts. For instance, Keegan & Bannister (2003) found that when a presenter wore attire that was color coordinated with their poster, more visitors came to the poster.

The flexibility that presentations afford also provides additional opportunities to make research memorable. For instance, while concrete words are remembered better than abstract ones, *memory for pictures is superior* ( Paivio & Csapo, 1969; Paivio & Csapo, 1973). Additionally, images that visualize information can convey more information, improving comprehension of the ideas they represent ( Robinson & Kierwa, 1995). Images can also be used more flexibly in presentations; for instance, images that are *bizarre* or humorous are remembered particularly well, likely due to a distinctiveness-based mechanism ( McDaniel & Einstein, 1986).

Kosslyn *et al.* (2012) provides additional presentation-related advice, grounded in psychological theory. Kosslyn has also published two books on this topic, providing a wealth of suggestions ( Kosslyn, 2007; Kosslyn, 2011).

## Further reading

I hope that grounding scientific writing advice in memory literature was useful in demonstrating the mechanisms whereby a research paper could be made more memorable. Nonetheless, I do not think this is a sufficient substitute for concise and well-written advice. In particular, I strongly suggest the reading of: Gray & Wegner (2013); Sand-Jensen (2007). Hengl and Gould’s brief guide to writing research entitled “Rules of thumb for writing research articles” is also a must-read.

For readers interested in additional advice on scientific writing and presenting: Kazdin (2013) provides a particularly well-written and concise summary of the necessary details to include when preparing a manuscript, as well as advice on selecting a journal and navigating the peer-review process. Dunleavy (2003) provides a comprehensive discussion of the scientific writing process and its related considerations. For further advice for poster presentations, see Block (1996) and “Creating Effective Slides Without Having to Become a Graphic Designer”. For more comprehensive guides on scientific presentations and speaking about science in public, see Albuquerque (2015); Alley (2013); and Meredith (2010).
