# Supplementary material for: Every scientist is a memory researcher: Suggestions for making research more memorable
Source: F1000Res. 2015 Jan 22;4:19. [Version 1] doi: 10.12688/f1000research.6053.1 (PMC4358409; doi:10.12688/f1000research.6053.1)
Supplement: Peer review history

# Peer review: Referee response for version 1

**DOI:** 10.5256/f1000research.6480.r7788

**Published:** 2015-03-09

**Authors:** Mathieu Hainselin

**Affiliations:** 1 Department of Psychology, Université de Picardie Jules Verne, Amiens, France

This paper is interesting for researcher, and I recommend its indexing with some possible modifications. I think, as a memory researcher, that this paper follows most of its own advice, and explains in a simple and concrete way how to improve manuscripts.

Although using all those advice is not easy, as the author writes himself, I think this paper would improve with some concreteness. For example, an example could be added after “With respect to scientific writing, if an amusing anecdote is used to help motivate the research question, the anecdote—as an emotional item—may be remembered well, but if too unrelated to the actual topic of the paper, the reader may later have trouble recalling where they read the anecdote.” or “Evidence also shows that imageability enhances association learning (Madan et al., 2010), which may make it easier to remember the links between different ideas within the paper later on. Providing illustrations of concepts (e.g., hypothesized results; theoretical models) may also help in this regard.” Likewise, less likely to be retained titles for this paper could help those who aren't (yet) memory researchers to see the differences between “good” and “bad” titles.

The sentence “In terms of scientific writing, a researcher likely reads many papers over the course of several months, and one that is distinctive and stands out from the rest is more likely to be subsequently remembered and later cited when the researcher is writing their own manuscript.” might be speculative. If there is no doubt concerning distinctiveness, the correlation between this memory effect and citations would need more references or some discussion.

More explicit explanations and examples concerning the repetition effect could also be interesting for the readers. Should authors write the same (main) message in the article?

In the end, despite minors remarks, the paper is very interesting and will probably be useful for many researchers.

I have read this submission. I believe that I have an appropriate level of expertise to confirm that it is of an acceptable scientific standard, however I have significant reservations, as outlined above.

# Peer review: Referee response for version 1

**DOI:** 10.5256/f1000research.6480.r7789

**Published:** 2015-02-24

**Authors:** Alan Castel

**Affiliations:** 1 Department of Psychology, University of California, Los Angeles, Los Angeles, CA, USA

This is an interesting and novel paper, with some very informative data!  I have two general comments 1) sometimes scientists are not good at knowing what is interesting/clear due to the curse of knowledge, and that could be tied into this paper (perhaps outsiders are better at choosing interesting titles or summarizing research findings?) 2) there is a strong metacognitive component regarding knowing what other people may or may not remember, and there is a lot of work showing that sometimes rememberers are not good judges of what is memorable.  The author talks about primary and recency effects but sometimes people even fail to incorporate serial position effects when making memorability judgments, albeit when studying lists of words (e.g., Castel, 2008), or fail to consider the effects that long retention interval have on memory (see much of Asher Koriat’s work), even when we might be explicitly aware of these issues in other contexts.  Suffice to say that people aren’t always the best judges of what is memorable, and this might also extend to titles of articles or how to make article memorable.  I think these two points could be considered, but overall I enjoyed reading this paper (and won't forget it)!

I have read this submission. I believe that I have an appropriate level of expertise to confirm that it is of an acceptable scientific standard.

# Peer review: Referee response for version 1

**DOI:** 10.5256/f1000research.6480.r7411

**Published:** 2015-02-09

**Authors:** Daniela Palombo

**Affiliations:** 1 Department of Psychiatry, Boston University, Boston, MA, USA

Dr. Madan has written an insightful and useful paper that can benefit writers in many fields of science. Grounding the paper's theme in memory phenomena is clever. I suggest the article be approved with reservations.

Here are my specific recommendations:  

Dr. Madan describes the "first mover advantage" (Newman, 2009): "a researcher publishing a modest paper on next year’s hot topic will accrue more citations than if they had instead published an outstanding paper on the current year’s topic." Dr. Madan suggests this may have to do with a primacy effect in memory. While this may be true, another obvious explanation for the "first mover advantage" is that the researcher simply misses the boat. Coming out before the wave means all those topical papers that will be published during the wave will cite you ---simply because it's a new topic and your paper is published and there may be little in the area. If you miss the wave, you miss out on those citations. So this is less attributable to memory but more about timing. This is another explanation that might be worth noting. 

Under "be interesting" I would stress the importance of transparency in writing a bit more. In my opinion, better remembered articles are those that I understood enough to be able to mention to other colleagues and explain to them fully. If an article has either too much technical jargon, is too brief, or is too complicated (as you note), it won't be fully comprehended and unlikely to be retained (and cited--no one wants to cite a paper they don't understand for fear you are not citing it accurately). Dr. Madan touches on these issues but I feel they can be emphasized more, as there is sometimes a tradeoff between sounding interesting and being clear. Both are important but one should not sacrifice the other.

I recommend after this sentence: "This phenomenon, known as the *peak-ends rule*, demonstrates that people’s memories of an experience are biased, with the ending being particularly important", that the author includes something a bit more concrete as a recommendation for capitalizing on recency. Perhaps it would be helpful for the author of a manuscript to summarize really clearly the main findings and implications of the study with a very powerful take-home message because that will be the last thing the reader remembers. I feel this is true for some of the other instances were the author draws on the memory literature in that more examples relevant to manuscript writing process would be helpful. 

The section on presentation specific advice is not quite as well developed as the section on manuscript writing. That is ok, in principle, since the paper's main focus is on written work. However, I do find I take less away from that part of the paper. I may consider beefing it up a bit. One recommendation I have regarding poster presentations is that they should be prepared with two types of visitors in mind: Visitor A is someone who sought the poster out and wants the full explanation from the presenter and will follow along on the poster as the presenter is speaking. Visitor B is someone who is moderately interested in the poster and wants to be able to get the "gist" from it in only a few minutes. The poster should stand on its own so that if Visitor B just wants a reprint they would be able to figure out what the study was about with fairly little effort.  

Otherwise, the paper sounds great.

I have read this submission. I believe that I have an appropriate level of expertise to confirm that it is of an acceptable scientific standard, however I have significant reservations, as outlined above.
